# Wireless Mouth Motion Recognition System Based on EEG-EMG Sensors for Severe Speech Impairments

**DOI:** 10.3390/s24134125

**Published:** 2024-06-25

**Authors:** Kee S. Moon, John S. Kang, Sung Q. Lee, Jeff Thompson, Nicholas Satterlee

**Affiliations:** Department of Mechanical Engineering, San Diego State University, San Diego, CA 92182, USA; sqlee@sdsu.edu (S.Q.L.); jthompson0878@sdsu.edu (J.T.); nsatterlee0532@sdsu.edu (N.S.)

**Keywords:** biomedical signal processing, wearable biomedical sensors, machine learning, speech disability, human–computer-interface

## Abstract

This study aims to demonstrate the feasibility of using a new wireless electroencephalography (EEG)–electromyography (EMG) wearable approach to generate characteristic EEG-EMG mixed patterns with mouth movements in order to detect distinct movement patterns for severe speech impairments. This paper describes a method for detecting mouth movement based on a new signal processing technology suitable for sensor integration and machine learning applications. This paper examines the relationship between the mouth motion and the brainwave in an effort to develop nonverbal interfacing for people who have lost the ability to communicate, such as people with paralysis. A set of experiments were conducted to assess the efficacy of the proposed method for feature selection. It was determined that the classification of mouth movements was meaningful. EEG-EMG signals were also collected during silent mouthing of phonemes. A few-shot neural network was trained to classify the phonemes from the EEG-EMG signals, yielding classification accuracy of 95%. This technique in data collection and processing bioelectrical signals for phoneme recognition proves a promising avenue for future communication aids.

## 1. Introduction

The ability to communicate with others is a fundamental human interaction; however, many individuals find this challenging due to damage to their vocal cords or other speech-impairing conditions. The complexity of recognizing silent speech has increased, but it remains challenging to improve the performance of real-time detection systems. Approximately 5.4 million Americans suffer from some degree of physical paralysis at present. The majority of these cases are due to strokes (34%), followed by spinal cord injuries (27%), individuals with a form of sclerosis (19%), and cerebral palsy (9%) [1]. In some instances, paralyzed individuals are unable to communicate through simple speech. People with paralysis must rely solely on the assistance of others to perform even the most fundamental communication tasks under these conditions. The purpose of this paper is to investigate a path that, on a very limited scale, could assist people with severe physical impairments in acquiring communication independence through a nonverbal interfacing system.

The problem faced here is that individuals with damaged vocal cords or other speech-impairing conditions are deprived of a fundamental human experience, the ability to communicate with others in their lives. By providing a solution to this problem, these individuals will be able to express their thoughts, hopes, and dreams and be able to fully live their lives through speech. Traditional methods of communication such as sign language, pen and paper, eye tracking, or other methods are adequate solutions. However, by tapping into the electrical signals of the body, these individuals are presented with another solution form which to choose from that has the potential to identify and communicate their thoughts more clearly.

The lips and tongue are the muscles directly connected to the brain which motivate researchers to exploit this potential for more complex tasks [2,3]. The amount of previous research on this topic is limited. Understanding the relationship between the brain and the specific mouth muscle movements is one of the primary objectives of this research. Broca’s area is essential in speech production. This area of the brain acts as a command center, orchestrating the complex muscle movements necessary for articulating spoken words. To form words and sentences, Broca’s area must relay signals to coordinate the muscles of the lips, tongue, and throat [4]. The studies establish a link between the left hemisphere and a specific stage of vocal development [5]. For instance, according to research, the superior temporal gyrus is most active during word perception, whereas Broca’s area is most active prior to speech articulation [6]. Interestingly, the motor cortex is the most active brain region during word and pseudoword articulation [7,8,9]. Finding and distinguishing the brain regions responsible for speech from those accountable for the motor functions of the tongue and lips are conducted to identify and locate the associated brain regions that control these movements and functions. The areas responsible for tongue and lip function appear slightly more prominent on both cortices. However, the premotor and motor cortex of the left hemisphere are believed to be the primary centers for tongue and lip movements [10].

This report explores the possibility of EEG (electroencephalogram) and EMG (electromyograph) data establishing communication for these individuals under speech-impairing conditions. EEG is a non-invasive method for measuring brain activity and a potentially useful technology for brain–computer interface applications. Multiple studies have examined how the tongue’s signal interacts with the brain. The motor cortex is the most active region of the brain, producing the most effective results at voltage frequencies between 70 Hz and 120 Hz (high gamma frequency) [11,12]. To distinguish the EEG signals while only moving the tongue, machine learning techniques were employed [13,14]. Various studies have implemented several data collection strategies to establish a connection between the brain and the tongue. This type of design comprises the user with a 3D-printed device that is lightweight and comfortable [15]. Another method of application involves wrapping a fabric strap around the head with embedded textile surface electrodes [16].

Although tongue movement can be detected from EEG signals, identifying patterns of tongue movement from the EEG signals is still challenging and can be difficult due to weak sensor signals, which require precise sensor data signal processing technology. In addition, wearability is crucial for obtaining EEG signals while moving [17,18]. Further, a study shows that EEG-measured brainwave frequencies can include facial muscle EMG signals [19,20]. EMG signals are used for detecting the electrical signals produced by the muscles. Incorporating EMG sensor input signals from mouth movement muscles can improve the performance of identifying mouth motions. The approach requires a real-time sensor network system and necessitates a sensor fusion model to acquire tongue movement from the different sensor types. This study proposes developing and employing a small and wearable EEG-EMG measurement system with wireless communication capabilities that is more practical in real-world settings.

This study also evaluates if neural networks could be trained on EEG and EMG data to accurately and efficiently recognize speech phonemes. This will be performed by collecting multiple EEG and EMG datasets of different mouthed phonemes from two different subjects, training neural networks that can ingest this information, potentially identify patterns in the data, and then accurately predict phonemes from test datasets. An LSTM (long short-term memory) network was initially trained on the dataset for phoneme classification. To increase accuracy, a few-shot CNN (convolutional neural network) was then trained on the data. Finally, augmentation was applied to the dataset and the few-shot CNN was again trained. This study explores the possibility of EEG (electroencephalogram) and EMG (electromyograph) data establishing communication for these individuals. Leveraging the capabilities of the neural networks, a system has been developed as a proof of concept aimed at recognizing phonemes, the basic units of human speech, from measured bioelectrical signals [21,22,23,24]. This approach involved collecting EEG and EMG data from the temples and jaws, respectively, associated with different phonemic sounds from various subjects.

This paper is structured as follows. The second section describes a custom-designed sensor system for concurrently sensing EEG-EMG signals with a set of biopotential electrodes that adhere comfortably to human skin. The third section explains the evolution of the correlation and covariance-based signal preprocessing method for extracting meaningful mouth motion patterns. The fourth section evaluates the ability of phoneme classification with neural networks. Lastly, the discussions and conclusions on the proposed method are provided.

## 2. Wearable Mouth Movement Monitoring System

**Figure 1:**
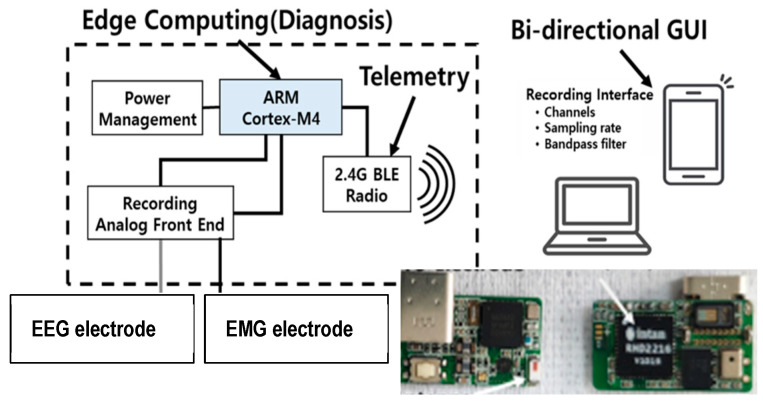
The circuit diagram of the wireless wearable mouth movement monitoring system.

We developed a novel EEG-EMG system that monitors mouth motion activities and provides rapid digital identification (Figure 1). The wearable wireless system employs biopotentials to monitor the EEG of brainwaves and EMG of facial muscles simultaneously [25]. In addition, the wearable sensor system contains a signal processing circuit that conducts edge computing before data transmission via a wireless data transmission chip. An external computer system is utilized for signal evaluation and feature categorization. The components of the sensor system are exhaustively described and specified in Table 1.

The precise placement of electrodes on the human head is critical to the design of this experiment. It has been clearly mentioned and emphasized that in order to achieve the best outcomes, a few particular regions of the head must be contacted with electrodes. To summarize, these sections include the left hemisphere of the head (for the EEG). A biopotential transducer translates brainwave and muscle movement data from the brain and chin to analog electrical signals. The signal acquisition module makes use of Intan Technologies, LLC’s digital electrophysiology interface chips (Los Angeles, CA, USA), which have a 4 kHz sampling rate per channel. The EEG and EMG signals were recorded using the RHD2216 chip, which is a low-power 16-channel differential amplifier paired with a 16-bit analog-to-digital converter (ADC). The wearable sensor communicates wirelessly via Bluetooth Low Energy (BLE). The signal is then wirelessly transmitted to a personal computer (PC), which uses MATLAB to categorize and process the signal data. The system captures sensor signals in real time, amplifies them, filters them, digitizes them, and wirelessly transmits them. Because of its low power consumption and flexibility, we picked the Nordic Semiconductor nRF52832 System-on-Chip (SoC) for computation and wireless data transmission on the module. For this experiment, a custom wearable sensor was built and constructed to serve as an EEG electrode, in addition to two commercially available adhesive patches.

## 3. Tongue Motion Recognition

### 3.1. Experimental Setup

**Figure 2:**
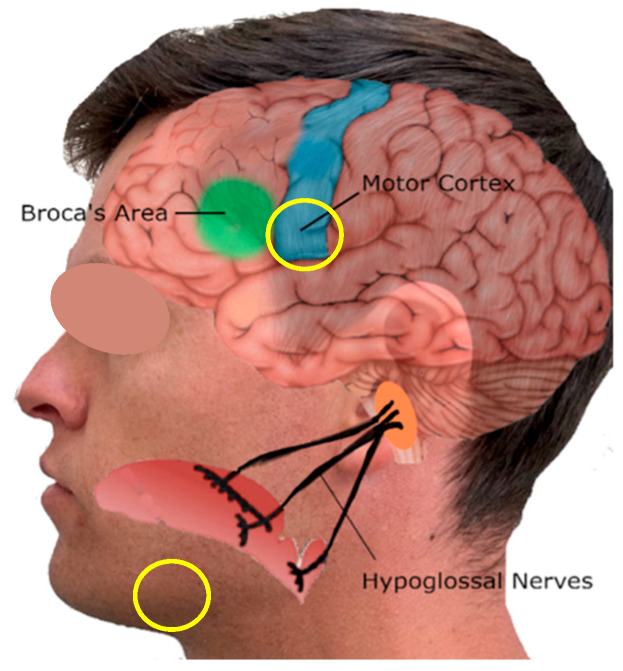
The wireless wearable tongue movement monitoring system.

The left hemisphere is the most active region of the brain when the tongue is activated, although the right hemisphere is also activated to a lesser extent [15,26,27]. In this experiment, the primary EEG sensor is positioned as close as feasible to the interior motor cortex in order to record brainwave signals from surface electrodes positioned near the center of activity on the tongue. Figure 2 depicts the intended positioning area for sensors.

Table 2 lists the experiments conducted. Experiment I, II, III, and IV depict a tongue motion pattern with slow and constant-speed movements. Multiple experiments were conducted to collect data using four electrodes and sensors placed at the previously mentioned locations. For this work, we collected experimental data without motion to avoid motion artifacts. The recordings were gathered and preserved on a laptop computer. Four commands were executed by the tongue in order to analyze information from the tongue muscle and brain and identify any patterns that may be present. According to previous research, the opening and closing of the eyes during EEG exams also affect the data in various ways [28,29]. Generally, it seems that opening the eyes during EEG investigations increases noise and interference in the data. Since the eyes are a crucial component of sensory reception, it follows that the brain receives a significant quantity of information while the eyes are open. This can affect brain activity in various regions involved in sensory reception [30]. Moreover, since blinking and closing the eyes is a natural and common occurrence, it seemed appropriate to investigate any effect that closing the eyes may have on the relationship between the tongue and the brain. Due to the fact that the state of knowledge in this area is still largely uncertain, each trial was conducted under eye-opening conditions.

Contact between the surface electrodes and the epidermis is a crucial consideration. Since the finest results are obtained when surface electrodes are held firmly against the skin, it is essential that they remain immobile during testing. As demonstrated in Figure 2, the target area for the surface EEG is a pubescent portion of the scalp, and the experiment must be designed so that hair does not need to be removed in order to wear this device. As the EEG surface electrodes are dispersed over an adhesive-coated material, this poses a design challenge. For the most precise results, it is essential that the sensor pad has exceptional skin contact. Although it would be effective to remove hair from the area in contact with the electrode pad, it would be impractical to mandate this for every participant in these EEG studies. Electrode material applied to the sensors provides a solution to this problem. This electrode gel is an electrically conductive medium that can be used between the sensors and the hair to eradicate the problem of hair’s poor conductivity. For the sensors, a specific gel (Spectra^®^ 360 Electrode Gel, ParkerLabs) is used to maintain resilient conductivity and minimal contact resistance. Due to the fact that the electrodes used in this experiment are two-channel surface electrodes, one sensor will be placed directly above the most caudal portion of the motor cortex, while the other will be placed just above the left ear, where there is little hair.

In Figure 2, a yellow circle depicts at the location where the second channel is installed. This area is sparsely haired but near the hypoglossal nerves that connect to the brain. The electrode gel is applied to both EEG sensors, which is secured by a fabric strap extending from the back of the head to the jaw. This should decrease the amount of translational movement between the sensors and the epidermis while permitting a hair-free contact area. Both sets of data from the two electrodes are collected and combined to provide a comprehensive view of brain activity.

### 3.2. Experimental Results

This section describes the outcomes of the methodologies described in the preceding section. The majority of the results are presented as graphs and charts to illustrate the data collected and analyzed from the wearable hardware and software. This section attempts to describe how the tongue and brain interpret sensor-collected signals, as well as the correlation patterns during directional tongue movements. Multiple sources demonstrate the tongue’s connection to the brain. It is well known that the motor cortex of the left hemisphere of the brain is the principal control center for the tongue. Despite the fact that multiple techniques and studies have demonstrated this to be true, little is known about the relationship between these two systems and how they can be utilized synchronously for nonverbal communication. Since the tongue can articulate multiple gestures using a complex group of muscles, it is essential to determine the relationship between brain activity and muscular activity as the tongue performs the various enumerated actions. In this section, the steps taken to analyze the data collected while the tongue performed a variety of movements are described. The tests were conducted with eyes open. This is to ensure normal wakeful brain activity within the frequency bands during data analysis, as it has been demonstrated that the alpha frequency range is substantially increased when the eyes are closed, and the brain is more meditative. Blinking was not regulated in the experiment.

Each trial consists of at least five repetitions of two- to five-second intervals, with a baseline between tongue movements during repose recorded between each repetition. The purpose of the EEG readings is to record and analyze data corresponding to the tongue activity being performed. For instance, if the tongue is elevated for three seconds, a distinct signal from the brain is acquired during those three seconds. The data points will then undergo multiple analyses to identify correlations between each movement. The purpose of this experiment is to correlate brain activity with EEG readings in order to eliminate interference from tongue and mandible movements. Due to the fact that previous research has indicated that EEG signals in this region of the brain are challenging to detect due to their low amplitude (on the V scale), careful attention must be paid during the experimental procedure and sensor placement in order to obtain the best possible signal. For the duration of these tests, the tongue was pressed with approximately 75% of its relative force capacity in an effort to maintain consistency across all experiments, although this was subjective to the person performing the activities.

**Figure 3:**
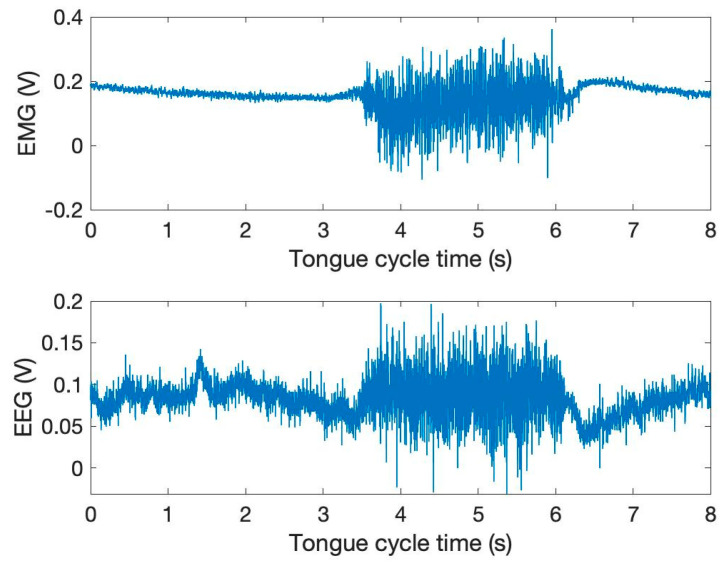
The time and amplitude characteristics of a typical EMG and EEG signal derived from the mandible and left-brain regions.

A series of experiments included several patients performing lingual tasks while EEG sensors were connected over a net across the scalp. Across the tests, the most substantial area of brain activity was in the left hemisphere towards the motor cortices [31]. Figure 3 illustrates the time and amplitude characteristics of a typical EMG and EEG signal derived from the mandible and left-brain regions. The illustration depicts the close coupling of EMG and EEG signals, as the synchronized graph patterns indicate [32,33,34,35]. The signals from the EEG and EMG sensors come from two separated wearables and also exhibit a normal distribution, as seen by the EEG channel’s attractive figure. A very similar histogram graph was also displayed by the EMG signal.

Figure 3 demonstrates the EMG and EEG activity as the tongue advances forward. There is a voltage fluctuation pattern visible in the diagram that is mirrored by both the tongue and the brain. The figure also demonstrates that the EMG and EEG signals are interconnected. The amplitude increase during tongue movement demonstrates dramatic variations. When the tongue is moved, there is consistent evidence of a shared pattern of voltage spikes across all experiments. 

**Figure 4:**
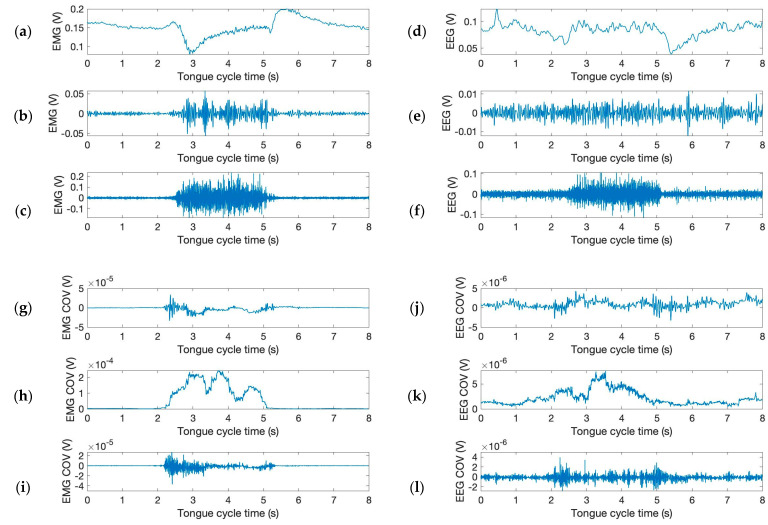
Moving window covariance calculated from the variation in EMG and EEG sensor signal. (**Upper**) The filtered time and amplitude signals from the mandible and left-brain locations: (**top**) (**a**,**d**) below 13 Hz, (**b**,**e**) between 13 and 30 Hz, and (**c**,**f**) above 30 Hz. (**Lower**) The time and covariance characteristics obtained from the graphs in (**a**–**f**): (**g**,**j**) below 13 Hz—between 13 and 30 Hz, (**h**,**k**) between 13 and 30 Hz—above 30 Hz, and (**i**,**l**) below 13 Hz—above 30 Hz.

The filtered (Butterworth) EMG and EEG signals of Figure 3 are shown in Figure 4a,b. Further, the moving window covariance calculates the variation in EMG and EEG sensor signal for each window. This study employs low (or “alpha”, below 13 Hz), high (or “gamma”, above 30 Hz), and mid (or “beta”, between 13 and 30 Hz) frequency ranges to filter EMG and EEG signals. The raw signals were also normalized to improve filtering performance. Figure 4a,b show the differences of the filtered signals between EMG and EEG signals indicating a low level of potential crosstalk if it exists. In Figure 4c,d, the moving covariances are also computed using a 500 ms data window and 2000 samples. Covariance is a quantitative measure of the simultaneous variability of two frequency bands. For instance, if larger values of one variable tend to correspond with larger values of another variable, this indicates positive covariance. Figure 4c,d depict the increased covariance outputs of alpha and beta between gamma frequencies during “spike”. The graph demonstrates that tongue movement affects increased power of gamma frequency band during “spike”. Clearly, covariance signals are synchronized with phases of tongue movement as signal heights increase during tongue motion. The majority of EEG activity seemed to be most prominent surrounding the time of the actual tongue movement, showing peak activities within roughly 2 s before and after. The high gamma activation range for tongue movement recording was between 70 and 120 Hz, with filters on the upper and lower end of the range in order to remove excess noise from various inputs [11].

**Figure 5:**
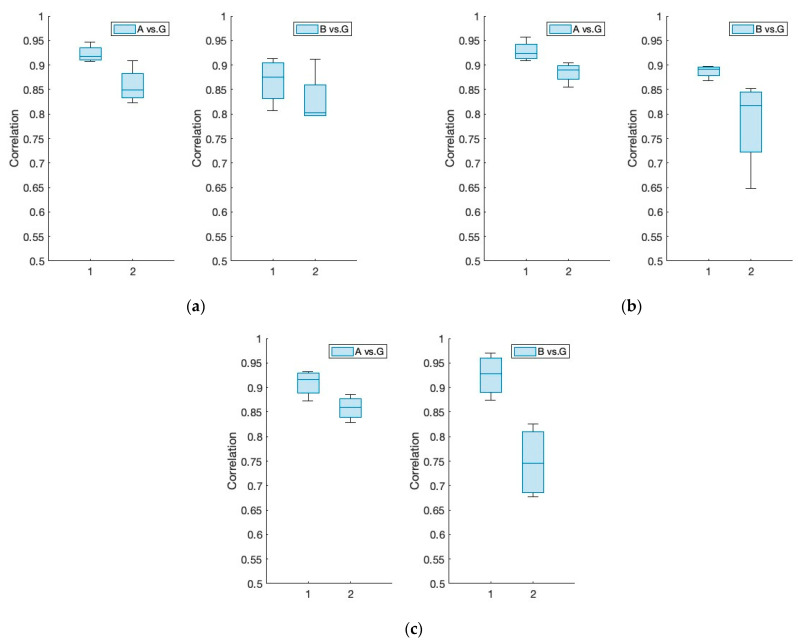
Correlation coefficient comparison between “pre” (1) and “spike” (2) in EEG sensor signal. (**a**) Forward; (**b**) right; (**c**) up: alpha (8–12 Hz), beta (13–30 Hz), and gamma (30–100 Hz).

Covariance is a function of the correlation coefficient. Thus, we conducted a correlation analysis to determine a statistical measure that indicates the strength of the relationship between the alpha, beta, and gamma frequency bands collected from the EEG. Comparing the “pre” datasets to the “spike” datasets, the calculated correlation coefficient data presented in Figure 5 provide evidence of the increased power of the gamma frequency band during the “spike”. As predicted, the ratio of frequencies reveals a greater proportion of alpha and beta frequencies during repose (or “pre”), which rapidly shifts to gamma frequency during tongue movement (or “spike”). This is consistent with previous research, which states that the gamma frequency increases when the brain is stimulated to perform an activity, as this is the frequency band most closely associated with intentional movement. This supports our hypothesis that tongue movement is related to EEG signals. When the tongue is active, both the musculature and the brain are stimulated to perform a task. Table 3 shows that the changes in the correlation coefficients during rest and stimulation not only provide a statistically roughly meaningful signal but also characterize the nature of this stimulation (i.e., the directional movements of the tongue).

In Table 3, the F-value is calculated as the ratio of group variation to within-group variation. A high F-value suggests that there is more variation across groups than within them. This shows that there is a statistically significant difference between group means (four samples each). The F critical value (5.987) is a specified figure to which the F-value can be compared to determine statistical significance. The table shows that the calculated F value for EEG signals is more than the F critical value (in the majority of cases), indicating a difference in correlation coefficients across groups. Furthermore, the estimated *p*-values are less than the 0.05 threshold employed in practice. The F statistic determines the *p*-value, which is the likelihood that the test results occurred by chance.

The correlation analysis provided insight to the behavior of frequency bands during tongue movements. In every experiment, correlation values and F values were strong (>0.7) between all frequencies. One of the most distinguishable patterns within the entire data sample was found in the correlation between alpha and gamma. In most cases, there was a decline in correlation during the spike phase of tongue movement. Since these two frequencies carry the most weight during rest and activity, it would follow that the correlation between the two decreases as the gamma frequency becomes more populated. Additionally, the correlation values and averages remained very similar between both samples with the eyes open and closed. While a few of the experiments do not directly mirror the other with the eyes open or closed, a key finding is that the correlation patterns remain largely the same when moving the tongue in certain directions.

This analysis provided insight into the nature of the alpha, beta, and gamma frequency bands and how they interact with each other during various tongue movements. When we observe the correlation change between two brainwave frequency bands differs significantly, we can deduce that brain power is required to move the tongue. We expect to see the most significant changes in the correlation between the alpha and gamma band, since these two frequencies are associated with opposite actions (rest and action, respectively). While this requires more investigation before it can be used in a practical manner, it allows insight into and understanding of this relationship.

## 4. EEG-Based Phoneme Recognition

EEGs, or electroencephalograms, are a noninvasive method of recording electrical activity in the brain. Of particular interest are its applications in brain–computer interfaces. By interpreting EEG signals, researchers are able to gain more information about the pathways and secrets of the brain by translating these micro-signals into usable information. EMGs, or electromyograms, on the other hand, measure muscle contraction electrical activity rather than brain electrical activity. This involves a different placement of electrodes either on the surface of the skin at the area of interest or a more invasive manner by inserting electrodes into muscles themselves.

In conjunction, these electrical measurement methods provide a new pathway for silent speech recognition. Damaged vocal cords or severe impairment such as ALS (amyotrophic lateral sclerosis), strokes, or traumatic brain injuries may result in a decreased ability to produce speech [36]. This would negatively impact an individual’s quality of life by stripping them of their ability of effective communication. EEGs are able to capture brain signals associated with specific speech phonemes, and when paired with the associated EMG muscle movement measurements in the jaw, speech can be predicated, and these patients can achieve a state of a silent communication.

### 4.1. Data Collection

**Figure 6:**
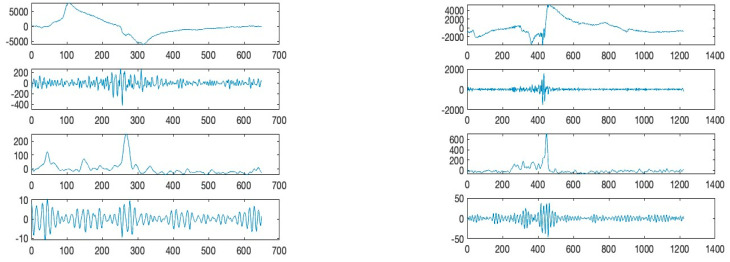
The time and amplitude characteristics of a typical EMG-contaminated EEG signal derived from the left-brain region (**left**: ‘D’ and **right**: ‘M’). From the top graph: (6-1) low-frequency component of EMG (<25 Hz); (6-2) high-frequency component of EMG (>25 Hz); (6-3) alpha and beta component of EEG; (6-4) gamma component of EEG.

To collect data for this experiment, the following process was taken. First, a list of English phonemes was made, namely M, D and A. Next, the training experiments were created. This was performed by recording an audio file that contained each single phoneme clearly pronounced a total of ten times, spaced apart by five seconds. This was followed by the entire word being clearly enunciated ten times at the end of the audio file. The purpose of this was to create a testing framework that could be played and followed by a test subject. The subject would repeat each phoneme as they were pronounced in the audio file; however, they would not utilize their voice box. In other words, they would be purely mouthing each phoneme so that the attached EMG-EEG electrodes would be able to capture the associated neural and electrical activity. Figure 6 illustrates the time and amplitude characteristics of a typical EMG and EEG signal derived from the mandible and left-brain regions. The illustration depicts the mixed nature of the EMG and EEG signals, as the synchronized graph patterns indicate.

From the raw sensor signals, the study uses a unique signal processing method that generates a combination of correlation and covariance patterns between different frequency bands of the EMG-contaminated EEG sensor signals. These patterns reflect the characteristics per sampling window for a selected combination of sample sensor signals (say, channels *X* and *Y*), and a set of correlations and covariances can be derived from a matching sampling window for the corresponding zone of channels *X* and *Y*.

The presented silent speech dataset contains a total of 60 samples for three phonemes, M, A, and D. The samples are collected from two different individuals, so 10 samples per person were recorded for each phoneme. Eight sets of signals were extracted from the 60 samples and used for training the machine learning models. Those eight sets are (6-1) low-frequency component of EMG (<25 hz); (6-2) high-frequency component of EMG (>25 hz); (6-3) alpha and beta component of EEG; (6-4) gamma component of EEG; (7-1) correlation of (6-1) and (6-2); (7-2) correlation of (6-3) and (6-4); (7-3) correlation of (6-1) and (6-3); (7-4) correlation of (6-2) and (6-4).

**Figure 7:**
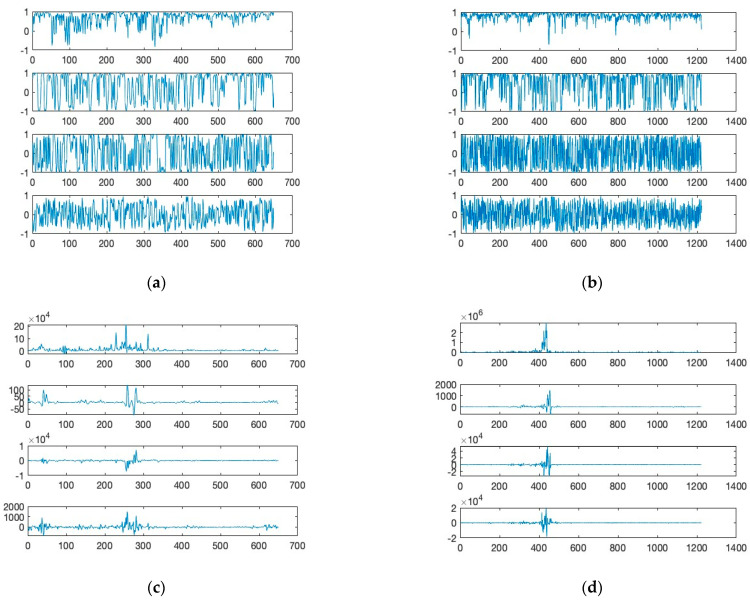
Moving window correlation and covariance calculated from the variation in EMG mixed EEG sensor signal. ((**a**,**c**) ‘D’ and (**b**,**d**) ‘M’). From the top graph, (**a**,**b**) (7-1) correlation of (6-1) and (6-2) of Figure 6; (7-2) correlation of (6-3) and (6-4); (7-3) correlation of (6-1) and (6-3); (7-4) correlation of (6-2) and (6-4). (**c**,**d**) (7-1) covariance of (6-1) and (6-2) of Figure 6; (7-2) correlation of (6-3) and (6-4); (7-3) correlation of (6-1) and (6-3); (7-4) correlation of (6-2) and (6-4).

In this following section, machine learning models, including LSTM and few-shot methods, were evaluated for efficacy on the collected covariance calculated from the EMG mixed EEG sensors from the brain area exhibiting discernible patterns, as shown in Figure 7. The goal of a neural network is to uncover some objective function from the dataset. Given sufficient high-quality data, the objective function can be estimated with high confidence. However, small datasets pose a specific challenge wherein the objective function is uncovered specifically for the training set, known as overfitting. Furthermore, part of the dataset must be reserved for validation, further reducing the training set and exacerbating the overfitting problem. To maximize the number of training samples, cross-validation is utilized with 10% of the samples reserved for the validation set.

### 4.2. The LSTM (Long Short-Term Memory) Neural Network

An initial study was performed with an LSTM (long short-term memory) neural network [37]. This type of neural network is generally used for processing sequential data due to a feedback mechanism where outputs are fed back into the network to update the network state. This work utilizes a two-layer LSTM network. The first layer takes a sequential input and produces a sequential output. The sequential output is fed into the second layer, which outputs only the last step the sequence. A fully connected layer then reduces the sequence to three outputs, one for each classification. A softmax layer then outputs the network results for each classification. The network is trained with the Adam optimizer, a learning rate of 0.0001, a batch size of 16, and a patience of 250 epochs (i.e., if loss does not decrease for 250 epochs, the training ceases). At the end of training, the epoch that yielded the lowest validation loss is used for accuracy assessment. 

The cross-validation study utilized 10% of the data (six samples) for the validation set and ten iterations. Each iteration uses two samples not previously evaluated, with the exception of the first sample of the M classification, which was used in the first and last iteration due to having one fewer sample than the other sets. Therefore, every sample in the dataset is included in the cross-validation study.

Samples were shuffled, and cross-validation on the entire dataset was performed three times. The best result yielded 68% accuracy. From a random classifier, 33% accuracy is expected, so while this is a marked improvement, the results are not within desired reproducibility for communication purposes. To determine if this is a data limitation or a model limitation, additional studies were performed. Specifically, there were techniques developed to increase accuracy on small datasets, including few-shot learning and applying data augmentation [38].

In addition, the network type was modified from an LSTM network to a standard CNN. The motivation for this is twofold. There is no evidence that an LSTM network outperforms a CNN in classification, and the LSTM is an order of magnitude slower since it requires feedback and thus cannot work in parallel [39].

### 4.3. Few-Shot Learning

To classify the data, a 1D convolutional few-shot neural network is employed. The few-shot network takes in an input sample and outputs a feature vector. To train the network, a set of reference samples from each class is passed into the network to obtain the reference feature vectors. As training samples are passed into the network, they are compared to the reference feature vector. The loss function forces training samples with the same label as a reference vector to move closer to the reference vector and samples with a different label to be pushed away from the reference vector during back propagation. This method of training is called contrastive loss. In this study, the cosine similarity function was employed to determine the similarity of the two feature vectors. The number of reference samples used for each class is the number of shots (e.g., three reference samples would be denoted as a three-shot network). In this study, a three-shot network is used in all cases unless otherwise noted. The reference samples are randomly selected four times in each case and those that yielded the smallest loss during training are selected.

**Figure 8:**
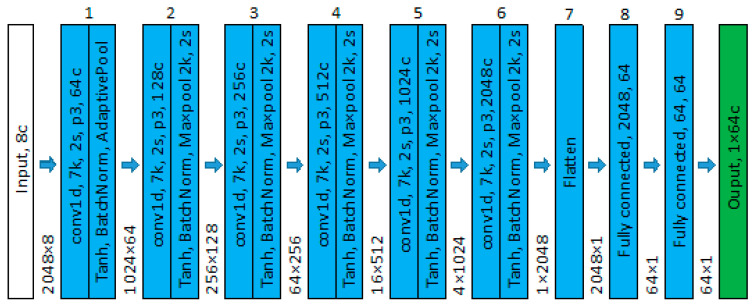
Network architecture for phoneme classification, where k is kernel, s is skip, *p* is padding, and c is channel. The adaptive pooling layer output in block 1 has a static output of 1024 to make the network invariant to input length. The tensor size is displayed between each of the blocks.

The network architecture is composed of nine blocks, as shown in Figure 8. The first six blocks (body) consist of 1D convolutions followed by tanh activation, batch normalization, and max pooling. The final three blocks (head) are a fully connected layer reducing the output to a feature vector of length 64. The network body gradually converts the 1D time series into channels via convolutional operations. The network depth allows features at any point in the time series data to be related to features at all other points. Furthermore, the fully connected head explicitly relates to all extracted features. The network was trained with a batch size of 16, a patience of 250 epochs, and a learning rate of 1 × 10^−4^ using the Adam optimizer. At the end of training, the epoch that yielded the lowest validation loss is used for accuracy assessment.

The three-shot network resulted in an 85% cross validation accuracy. This presents a significant increase over the LSTM results. However, higher accuracies are still desirable for communication applications. Therefore, the impact on augmenting the dataset is also explored.

To increase the variance of the training set and reduce overfitting, augmentation on the training data is employed. Augmented operations are randomly applied to the training set only each time a sample is accessed. Augmentation can be performed on the entire sample and/or sections of the samples. Entire sample augmentation includes adding a random slope to the sample, shifting the sample in the x-direction, shifting the sample in the y-direction, and scaling the sample. Sections of the sample are augmented by defining a moving window over the sample and applying an operation to the overlapping area with a low probability. These augmentations include hanging datapoint, Gaussian filter, noise addition, and downsampling. All augmentations are applied in random order. These types of augmentations are also selected to improve the model by making it robust to voltage shifts, noise intrusions, and brief recording errors. In addition, random cropping is performed with the intent of promoting weaker features to improve classification.

The results of the augmentation on the three-shot model show a large improvement of 95% accuracy. Only three signals (two D and one M) were misclassified. It is expected that these misclassifications may be corrected and higher accuracies can be achieved with further training samples.

## 5. Discussion

The purpose of this study was to develop a small and wearable EEG-EMG measurement system and data analytics for silent speech recognition. To support the study aim, we studied the relationship between the brain and the tongue muscles responsible for directional movements for potential nonverbal communication. In a series of experiments, EEG and EMG were used to record muscle and brain activity with a multimodal wearable sensor as the tongue was routinely moved forward, right, and up while at rest. To record electrical activity during these actions, two EEG sensors were affixed to the head near the left temporal lobe, just above the left ear, and two EMG sensors were attached underneath the chin, near the large inner section of the glossal muscles. Then, a correlation and covariance analysis was conducted to determine the important characteristics of the collected data.

The EMG transducer detects both low and high frequency movements. Similarly, this technique divides the EEG signal into three frequency bands: alpha (8–12 Hz), beta (13–30 Hz), and gamma (30–100 Hz). In fact, the proposed feature extraction methods, which use correlations and covariances between filtered biopoetential signals, are a unique approach with few comparable publications. We observed that the changes between the two signals, specifically the covariance change in the signals, may be used to evaluate the relationship between the two signals and how much they change together. The results indicated that the activities of the brain and tongue were distinct and suggested that, with a larger sample size, they could be distinguished. The analysis of correlation (and covariance) revealed patterns between the alpha and gamma frequencies that enabled the detection of tongue movements.

A possibility that should not be overlooked is that there is contamination in the EEG samples due to cross-talk between sensors or even unwanted input from various muscles around the head, specifically around the area of the EEG sensor location. If this were the case, then the analysis performed would have little meaning since most of the EEG data would merely reflect muscular activity. However, further analysis suggests that these data are not a product of cross-talk or input from other muscles. The voltage received from EEG studies is consistently lower than the EMG voltage, which allows a closer look at the hyperpolarization period after activity. This period does not mimic the pattern of EMG voltage spikes and can be considered unique to EEG readings. The literature review has suggested that the alpha, beta, and gamma bands within this physical region are linked to wakeful activity and should reflect that within each frequency range. The resulting table highlights this change, showing how each frequency composition is different when the brain is being used to move the tongue versus when it is at rest. These findings imply that the EMG and EEG readings are, in fact, unique and not a product of cross-talk or interference from other muscles or external sources. 

While these findings that have been presented can be considered significant, there is much left to be understood behind the physiological mechanism that allows the brain to control the tongue in a practical, noninvasive manner. This discovery, coupled with the correlation (or covariance) study, provides a much clearer understanding of the brain and how it controls tongue movements. Future research for this study would benefit from the use of a neural network with a significantly larger sample size. While trends were present within the analyses performed, it would be beneficial to perform similar tests on multiple test subjects rather than just one as seen here. More prominent trends in data would be more prevalent over a larger scale. Additionally, electrodes placed over the left temporal lobe will have interference from the hair on the head, regardless of the aid of electrode gel. A shaved head would increase the accuracy of the sensors, as well as reduce noise within the EEG. Lastly, as the EEG and EMG were not technically utilized synchronously, future studies should look to implement a combination of post processing between both studies to ensure maximum accuracy among directional tongue movements.

This research can be furthered in various ways. While this experiment only used EEG data, EMG data were also collected. In efforts to increase accuracy, these EMG data could be incorporated and processed in tandem with EEG data so that multiple data origins can be used in phoneme recognition. This path would likely be more difficult since these data sources produce different types of data that would need to be collected, normalized, and processed to allow them to both be used in neural net training. The ultimate goal would be to have a single trained neural network that is able to recognize any phoneme based on EEG/EMG data. Therefore, we performed a comparison on various machine learning models for phoneme classification.

Neural network training on the phoneme dataset yielded promising results, with accuracies displayed in Table 4. Due to the small dataset, a few-shot method with contrastive loss was found to outperform an LSTM network with softmax classification. The reason for this improvement is due to direct comparison of extracted features for classification. In addition, CNN networks train faster than LSTMs and thus optimal hyperparameters can be selected more easily. Further accuracy increases were observed by augmenting the dataset with 95% accuracy obtained during cross-validation. 

While the few-shot model is capable of handling samples of variable length, it is unable to classify more than one phoneme in a signal. This means that phonemes must be segmented prior to classification. To overcome this challenge, a more sophisticated model such as a sequence-to-sequence transformer model can be employed. Such models are used to transcribe audio to text in real time but require large datasets. However, practical challenges in data collection with EMG-EEG (e.g., attaching sensors) has been a substantial barrier to obtaining large quality datasets. Therefore, given the simple nature of the proposed device and the high accuracy achieved in this preliminary study, further data collection can be more easily performed to facilitate future research.

## 6. Conclusions

To conclude, the results found within this study provide greater insight into the pathway from the brain to the tongue and allow future research to utilize these principles to create a noninvasive system that can use the brain and tongue simultaneously to create a user-controlled device. This study intends to demonstrate the viability of generating distinctive EMG and EEG patterns with tongue motions to recognize different directional movements utilizing a novel wireless EMG and EEG wearable network technique. A wireless EMG-EEG sensor system that permits quick and extremely sensitive data collection and a machine learning algorithm for tongue motion-based detection from the EMG-EEG sensor signals make up the study’s two primary components.

Although research has found various methods of utilizing tongue movements to create human–machine interfaces, many of these methods employ invasive techniques that are either uncomfortable or impractical for long-term use outside of controlled environments. The research methods included in this study will use surface electrodes placed at specific locations around the head to record voltage patterns from the tongue and the brain during specific tongue movements. An electroencephalograph (EEG) will measure the voltage output from the brain through surface electrodes coupled with electrode gel, while an electromyograph (EMG) will record the voltage gathered from the tongue muscles under the jaw with dry surface electrodes. Modern, higher-quality electrodes can also be used to record voltage for future study. Although the results suggest a strong possibility of achieving this, further exploration and research is required to fully develop a functional nonverbal communication device for those who are vocally impaired.

In conclusion, this technique of phoneme recognition proved successful in accurately identifying phonemes from EEG and EMG data. The neural networks are able to take the raw data from the three recorded phonemes, and while having different patterns, this network is able to identify patterns within the phonemes and provide accurate recognition. Once data from test subjects of multiple genders were combined, validation accuracy drastically increased. This concludes that the temple area of the human–machine interface is a viable area for phoneme recognition when no sound is made. This opens up an opportunity for silent communication; however, there is still remaining research that needs to be performed to expand this to phonemes mixed in words and not only a single phoneme. This next step will require real-time neural net modeling to be able to process this information in real time.

## 7. Patents

The following patent is partially resulting from the work reported in this manuscript: Moon, K.S., Lee, S.Q. An Interactive Health-Monitoring Platform for Wearable Wireless Sensor Systems. PCT/US20/51136.

## Figures and Tables

**Table 1:** The specifications of the wireless wearable sensor.

Specification	Description	Value
Power source	Rechargeable battery	8 h/charging
Data transmission	BLE 5.0	1 M bps in 2 m
EEG/EMG electrodes	Disposable Ag/AgCl standard, pre-gelled and self-adhesive	(20 × 20) mm
Front-end circuit	Intan Tech Chip (RHD2216)	10 mV, 16 bit, 16 ch
Onboard CPU	ARM Cortex M4	4096 Hz/ch sampling rate
Wireless circuit	NRF 52X	2.4 GHz, BLE 5.0

**Table 2:** A breathing cycle experimental protocol.

Experiment	Sampling Time
I—Up motion	3 s
II—Right motion	3 s
III—Forward motion	3 s
IV—Neutral position	3 s

**Table 3:** A F-value and *p*-value table for correlation coefficients obtained from EEG.

Tidal Volume	F-Value (Forward)	*p*-Value (Forward)	F-Value (Right)	*p*-Value (Right)	F-Value (Up)	*p*-Value (Up)
Alpha vs. Gamma	10.07	0.0192	8.71	0.0256	7.43	0.0344
Beta vs. Gamma	1.22	0.3119	4.78	0.0715	17.25	0.006
Critical value	5.987	0.05	5.987	0.05	5.987	0.05

**Table 4:** Comparison of classification accuracy from each neural network tested.

Model	LSTM	Few-Shot	Few-Shot + Augmentation
Accuracy	68%	85%	95%

## Data Availability

The original contributions presented in the study are included in the article, further inquiries can be directed to the corresponding authors.
